# Probing conformational dynamics to understand kinase inhibition

**DOI:** 10.7554/eLife.92753

**Published:** 2023-10-18

**Authors:** Ian R Outhwaite, Markus A Seeliger

**Affiliations:** 1 Department of Pharmacological Sciences, Stony Brook University Stony Brook United States

**Keywords:** ERK2, MAP kinase, inhibitor, allosteric regulation, NMR, hydrogen-deuterium exchange mass spectrometry, None

## Abstract

Why do some inhibitors select the on-state in ERK2, a kinase that is involved in many signaling pathways in cells, whereas others bind to more than one conformation?

**Related research article** Anderson JW, Vaisar D, Jones DN, Pegram LM, Chen H, Moffat JG, Ahn NG. 2023. Conformation selection by ATP-competitive inhibitors and allosteric communication in ERK2. *eLife* **12**:RP91507. doi: 10.7554/eLife.91507.

Protein kinases are enzymes that act as molecular switches for signaling pathways in cells. Most kinases have an “on”-state that promotes signaling, and an “off”-state that does not, and these two states have different shapes or conformations. A number of diseases are associated with kinases not functioning properly, so kinases are important targets for researchers working in drug development.

In general, when a protein kinase is switched on, ATP (a molecule that provides cells with energy) binds to the active site of the kinase, and the kinase facilitates the transfer of a phosphate group from ATP to a protein. This means that it is possible to inhibit a kinase by getting a small molecule to bind to the active site instead of ATP. Some of these inhibitors can bind to just one state, but others can bind to both the on-state and the off-state.

To develop better drugs it is important to understand how kinase conformations relate to signaling and how inhibitor molecules select for these conformations. Now, in eLife, Natalie Ahn (University of Colorado, Boulder) and colleagues – including Jake Anderson as first author – report the results of experiments on a kinase called ERK2 that is involved in many signaling pathways in cells (Anderson et al., 2023). ERK2 is of particular interest because it is a key player in the MAPK signaling pathway that is often misregulated in human cancers (Roskoski, 2019; Lavoie et al., 2020).

In many kinases the on-state and the off-state have very different confirmations, but that it not the case for ERK2 (Zhang et al., 1994; Canagarajah et al., 1997). Indeed, the difference is so subtle that it is difficult to distinguish between the two states with X-ray crystallography, a technique that is widely used to determine the static structures of biomolecules. Fortunately, the dynamics of the two states are different, so Anderson et al. were able to use two established techniques for the study of dynamics – nuclear magnetic resonance and hydrogen-deuterium exchange mass spectrometry – to examine the dynamics of ERK2. They found that the the on-state corresponds to at least two conformational substates (named R and L) that interconvert on a timescale of milliseconds.

First, the researchers – who are based the University of Colorado in Boulder, the University of Colorado Anschutz Medical Center and Genentech – tested the impact of three inhibitors on the ERK2 kinase. They found ERK2 is predominantly in the R-state after it binds to an inhibitor called VTX11e, and similarly when it binds to BVD523. However, when ERK2 binds to an inhibitor called GDC0994, it remains in both the R-state and the L-state. What chemical motifs in VTX11e and BVD523 select for the ERK2 R-state? In an efort to answer this question the researchers tested a series of 17 ERK2 inhibitors that were similar to GDC0994 in various ways (see Figure 3 of Anderson et al., 2023). For three of these inhibitors the ERK2 kinase remained in both the R-state and the L-state, but in thirteen cases it selected the R-state (similar to the behavior observed for VTX11e and BVD523.) The remaining inhibitor did not fall neatly into either category.

To better understand the mechanism underlying such selection, Anderson et al. determined the three-dimensional structure of several inhibitors bound to ERK2. They observed that the large bulky chemical group on the right side of GDC0994 may have prevented conformational selection of the R-state, as it did for other inhibitors. The three inhibitors for which the ERK2 kinase remained in both the R-state and the L-state also had similar bulky chemical groups on their right sides, whereas the thirteen inhibitors that selected the R-state did not (Figure 1A). This observation prompted the researchers to ask what would happen if the same bulky group was added to the right side of an inhibitor that would normally select the R-state of ERK2. Luckily, an inhibitor called ATG017 has such a structure, and Anderson et al. found that it was able to bind to both the R-state and the L-state of ERK2, just as GDC0994 does (Figure 1B). This is consistent with previous work on ATG017 (also known as AZD0364) by AstraZeneca (Ward et al., 2019; Flemington et al., 2021).

**Figure 1.**
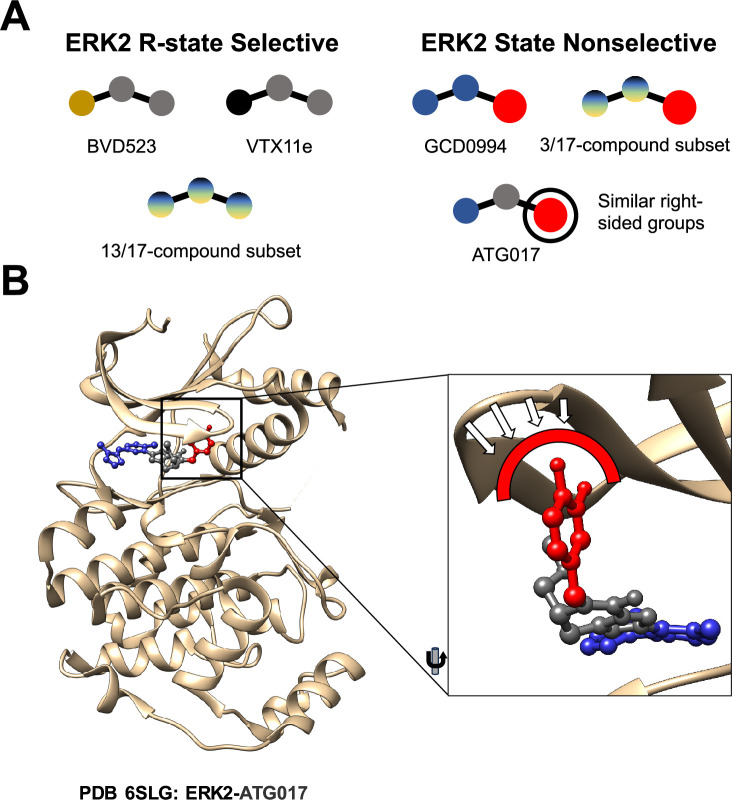
Comparing different ERK2 inhibitors. (**A**) BVD523, VTX11e and 13 of the 17 ERK2 inhibitors studied by Anderson et al. select for the R-state when binding to the ERK2 kinase. GCD0994, ATG017 and 3 of the 17 inhibitors do not select for the R-state: these inhibitors have similar structures, with a bulky chemical group (red circle) on their right side. (**B**) A close-up view of ATG017 (red/grey/blue) bound to ERK2 (beige). With most inhibitors the Gly-loop of ERK2 can bend downwards into a highly stable closed position that is associated with the R-state. With ATG017, however, the bulky chemical group (red) prevents the Gly-loop from reaching this position, so ERK2 is free to assume more than one conformation.

The work of Anderson et al. also sheds light on how the spatial configuration of atoms in ERK2, the inhibitors and even the surrounding solvent contribute to promoting these conformational preferences. For example, when an R-state-selective inhibitor binds to ERK2, movements in the upper lobe of the kinase bring the residues required for catalytic activity closer together. Furthermore, ERK2 is regulated in an allosteric manner (that is, the regulatory site and the active site are different), and Anderson et al. show that the residues involved in allosteric regulation re-arrange their positions in order to relay an allosteric signal from the regulatory site to the active site. Additionally, certain R-state-selective inhibitors influenced the flexibility of the activation loop of ERK2, which prompted Anderson et al. to suggest that these inhibitors might also influence how ERK2 is regulated by other proteins.

In summary, Anderson et al. show how small changes in ERK2 inhibitors might lead to larger changes in ERK2 dynamics. Specifically, their work provides a roadmap for how to engineer and study compounds that preferentially bind the ERK2 kinase in the R-state. Moreover, it will be interesting to explore if comparable phenomena occur with other similar kinases.

## References

[bib1] Anderson JW, Vaisar D, Jones DN, Pegram LM, Chen H, Moffat JG, Ahn NG (2023). Conformation selection by ATP-competitive inhibitors and allosteric communication in ERK2. eLife.

[bib2] Canagarajah BJ, Khokhlatchev A, Cobb MH, Goldsmith EJ (1997). Activation mechanism of the MAP kinase ERK2 by dual phosphorylation. Cell.

[bib3] Flemington V, Davies EJ, Robinson D, Sandin LC, Delpuech O, Zhang P, Hanson L, Farrington P, Bell S, Falenta K, Gibbons FD, Lindsay N, Smith A, Wilson J, Roberts K, Tonge M, Hopcroft P, Willis SE, Roudier MP, Rooney C, Coker EA, Jaaks P, Garnett MJ, Fawell SE, Jones CD, Ward RA, Simpson I, Cosulich SC, Pease JE, Smith PD (2021). AZD0364 Is a potent and selective ERK1/2 inhibitor that enhances antitumor activity in *KRAS*-mutant tumor models when combined with the MEK inhibitor, Selumetinib. Molecular Cancer Therapeutics.

[bib4] Lavoie H, Gagnon J, Therrien M (2020). ERK signalling: A master regulator of cell behaviour, life and fate. Nature Reviews Molecular Cell Biology.

[bib5] Roskoski R (2019). Targeting ERK1/2 protein-serine/threonine kinases in human cancers. Pharmacological Research.

[bib6] Ward RA, Anderton MJ, Bethel P, Breed J, Cook C, Davies EJ, Dobson A, Dong Z, Fairley G, Farrington P, Feron L, Flemington V, Gibbons FD, Graham MA, Greenwood R, Hanson L, Hopcroft P, Howells R, Hudson J, James M, Jones CD, Jones CR, Li Y, Lamont S, Lewis R, Lindsay N, McCabe J, McGuire T, Rawlins P, Roberts K, Sandin L, Simpson I, Swallow S, Tang J, Tomkinson G, Tonge M, Wang Z, Zhai B (2019). Discovery of a potent and selective oral inhibitor of ERK1/2 (AZD0364) that is efficacious in both monotherapy and combination therapy in models of nonsmall cell lung cancer (NSCLC). Journal of Medicinal Chemistry.

[bib7] Zhang F, Strand A, Robbins D, Cobb MH, Goldsmith EJ (1994). Atomic structure of the MAP kinase ERK2 at 2.3 A resolution. Nature.

